# diaPASEF Proteomics and Feature Selection for the Description of Sputum Proteome Profiles in a Cohort of Different Subtypes of Lung Cancer Patients and Controls

**DOI:** 10.3390/ijms23158737

**Published:** 2022-08-05

**Authors:** María del Sol Arenas-De Larriva, Alejandro Fernández-Vega, Bernabe Jurado-Gamez, Ignacio Ortea

**Affiliations:** 1 Pneumology Department, Reina Sofia University Hospital, Maimonides Biomedical Research Institute of Cordoba, University of Cordoba, 14004 Cordoba, Spain; 2 Institute for Biomedical Research and Innovation of Cadiz (INiBICA), 11009 Cadiz, Spain; 3 Proteomics Unit, CINN, CSIC, Instituto de Investigación Sanitaria del Principado de Asturias (ISPA), 33011 Oviedo, Spain

**Keywords:** lung cancer, sputum, proteomics, diaPASEF, adenocarcinoma

## Abstract

The high mortality, the presence of an initial asymptomatic stage and the fact that diagnosis in early stages reduces mortality justify the implementation of screening programs in the populations at risk of lung cancer. It is imperative to develop less aggressive methods that can complement existing diagnosis technologies. In this study, we aimed to identify lung cancer protein biomarkers and pathways affected in sputum samples, using the recently developed diaPASEF mass spectrometry (MS) acquisition mode. The sputum proteome of lung cancer cases and controls was analyzed through nano-HPLC–MS using the diaPASEF mode. For functional analysis, the results from differential expression analysis were further analyzed in the STRING platform, and feature selection was performed using sparse partial least squares discriminant analysis (sPLS-DA). Our results showed an activation of inflammation, with an alteration of pathways and processes related to acute-phase, complement, and immune responses. The resulting sPLS-DA model separated between case and control groups with high levels of sensitivity and specificity. In conclusion, we showed how new-generation proteomics can be used to detect potential biomarkers in sputum samples, and ultimately to discriminate patients from controls and even to help to differentiate between different cancer subtypes.

## 1. Introduction

Lung cancer is the neoplasm with the highest mortality rate worldwide [1]. Smoking is the main risk factor for lung cancer, and due to the increase in female smokers in recent years, the incidence of the disease is expected to rise [2]. Histologically, adenocarcinoma—which originates in the glandular cells of the lungs—is the most common form of lung cancer, whereas squamous, large- and small-cell carcinoma rates have been decreasing [2,3]. Although great advances in diagnosis, surgical techniques and pharmacological treatment have been introduced in recent years, the average 5-year survival rate remains at 10–15% [1], mainly because diagnosis usually occurs at an advanced stage of the disease. The survival rate exceeds 70% in stage I patients, although in more than 60% of cases, the diagnosis is made at advanced stages [1]. Therefore, the delay in diagnosis and tumor extension are responsible for the high mortality rate in lung cancer. The high mortality, the presence of an initial asymptomatic stage, and the fact that diagnosis in early stages reduces mortality justify the implementation of screening programs in at-risk populations.

Several procedures are being used in the screening and diagnosis of lung cancer, mainly chest X-ray, computed tomography (CT) and positron emission tomography (PET). Radiographic studies have the disadvantage of a high false negative rate due to occult or small-cell lung cancer. In fact, the National Lung Screening Trial (NLST) study has shown a 20% reduction in mortality from lung cancer in at-risk populations when low-dose computed tomography (LDCT) is used instead of chest radiography [4]. In the USA, the Preventive Services Task Force revised the guidelines on lung cancer screening in 2013 [5]. It recommends annual screening with LDCT for high-risk smokers and ex-smokers (age 55–80 years; cumulative consumption of 30 packs/year or ex-smokers who have quit within the last 15 years). However, LDCT screening for lung cancer has a low specificity: in the NLST study, of the 39% of participants in whom a lung lesion was observed by LDCT, 95% showed false-positive results [6,7]. In the management of indeterminate pulmonary nodules discovered by CT, two strategies are used: non-invasive techniques based on radiological follow-up, and invasive techniques based on biopsy to obtain material for cyto-histological study. The non-invasive approach has certain drawbacks for the patient, such as the extra radiological exposure and the anxiety generated by the procedure. Additionally, the large additional cost of CT monitoring of the entire at-risk population (approximately 25% of the population; active smokers or ex-smokers at risk) has an impact on the sustainability of public health systems. On the other hand, biopsy is a more aggressive method that also carries certain risks, such as pneumothorax, hemorrhage and false negatives. Therefore, it is imperative to develop less aggressive methods that can complement LDCT for the diagnosis of lung cancer so that it can be incorporated into the screening program, improving its cost-effectiveness.

In this sense, the use of omics disciplines, mainly proteomics and metabolomics, to identify markers is one of the most promising lines of research [8]. Thus, the use of volatile and non-volatile compounds in exhaled air with diagnostic or prognostic capabilities has been extensively studied [9]. Our group is also a pioneer in the study of the applicability in lung cancer of compounds present in sweat [10,11]. Recently, using proteomics, we studied the mini bronchoalveolar lavage fluid (mini-BALF) obtained by bronchoaspirate [12]. Mini-BALF is a minimally invasive endoscopic technique commonly performed in the study of lung cancer. It collects fluid from alveolar and bronchial sections and is, therefore, very close to the nodule or tumor. The soluble proteins present in BALF are plasma-derived or secreted from bronchial epithelium and immune cells [13]. BALF proteins have been reported as potential biomarkers in several lung diseases, such as idiopathic pulmonary fibrosis [14], chronic obstructive pulmonary disease (COPD) [15], and hypersensitivity pneumonitis [16]. In our BALF study, we report a panel of potentially biomarker proteins to differentiate between lung adenocarcinoma patients and control subjects [12].

On the other hand, sputum is a readily available fluid containing exfoliated lung airway epithelial cells, and its cytological study can detect morphological alterations, although with low sensitivity [17]. The use of omics technologies has started to be applied to sputum, and the first biomarkers have been proposed. Thus, using metabolomics and genomics techniques, it has been reported that the amount of certain lipids [18] and two microRNAs [19] varies in the sputum of lung cancer patients compared to controls. Proteins present in sputum have also been studied, using proteomics techniques, with the aim of discovering possible biomarkers in COPD [20,21] and in lung cancer [22,23]. Yu et al. [22] described a differential abundance of ENO1 in the sputum of patients with lung cancer using ELISA, although with low sensitivity and specificity. Ali-Labib et al. [23] found an increase in sputum protein MMP2 in lung cancer. Both studies are too small to generalize the results, so they must be verified and validated.

Mass spectrometry (MS)-based proteomics has made a quantum leap in quality in recent years due to a combination of advances in instrumentation, sample preparation methodologies and computational analysis [24]. Thanks to these multiple advances, these new technologies have come to be called next-generation proteomics, to reflect their ability to characterize virtually complete proteomes [25]. Some of the most significant recent advances are data-independent acquisition (DIA), ion mobility, and parallel accumulation-serial fragmentation (PASEF) [26], which together allow the routine, reproducible and highly efficient quantification of proteomes at a much greater depth. DIA also allows the rapid conversion of a small amount of tissue or body fluid into a unique, permanent digital file representing the proteome of the sample [27]. These omics maps can then be further analyzed, re-analyzed, compared and interrogated in silico to detect and quantify proteins in multiple samples. DIA technology is already being applied in clinical research in cancer, insulin resistance, cardiovascular disease and Alzheimer’s disease [28].

In this study, we aim to identify sputum proteins that can classify, within our cohort of patients, individuals with lung cancer, and to build a classification model with potential clinical utility based on the panel of proteins that identify lung cancer patients. From this model, different analytical methods could be developed that can be implemented in clinical biochemistry laboratories for the diagnosis and/or screening of at-risk subjects with a higher probability of having lung cancer.

## 2. Results and Discussion

Seventy-two individuals were recruited, 47 cases and 25 controls. After studies using the anatomical pathology department, the 47 lung cancer patients were divided into the following subtypes: 17 adenocarcinoma, 11 squamous, 15 microcytic, and 4 carcinoma NOS (not otherwise specified). After obtaining sputum samples from these individuals, a proteomics study was performed for the massive quantification of the proteins detected in these samples. This analysis was performed using nLC–MS-based shotgun proteomics with diaPASEF acquisition. diaPASEF windows were optimized as described in the Material and Methods section, making a total of 64 windows (Supplementary Table S1). To process the diaPASEF runs, we used the directDIA workflow in Spectronaut software, which is based on an initial spectrum-centric search of the DIA data, to make a sample-specific library that was then used for a peptide-centric search of the data. Therefore, this workflow has the advantage over a traditional DIA quantification workflow in that there is no need to build an ad hoc library from the samples, thus avoiding the need for prior LC–MS DDA runs.

Our analysis resulted in the identification of a total of 552 protein groups, corresponding to 914 proteins, and 527 protein groups quantified (Supplementary Table S2), with an overall protein group FDR of 1%. Considering the 527 protein groups quantified, missing data represented only 0.04% (15 data points out of a total number of measurements of 40,052), showing the high degree of completeness achieved by the workflow followed. As an indicator of the quantitative accuracy of the workflow, the experimental CVs of the areas quantified for each protein group were calculated for five technical replicates of one of the samples. The median CV for all the protein groups quantified was 23.4%. This value is higher than what is usually observed in benchmarking studies that use standards or cell cultures as a sample source [29], but is in line with that obtained in biological fluids, where the complexity of the sample in terms of dynamic range and composition causes the quantification accuracy to be lower.

### 2.1. Proteome Changes in the Sputum Proteome of Lung Cancer Patients

**Figure 1:**
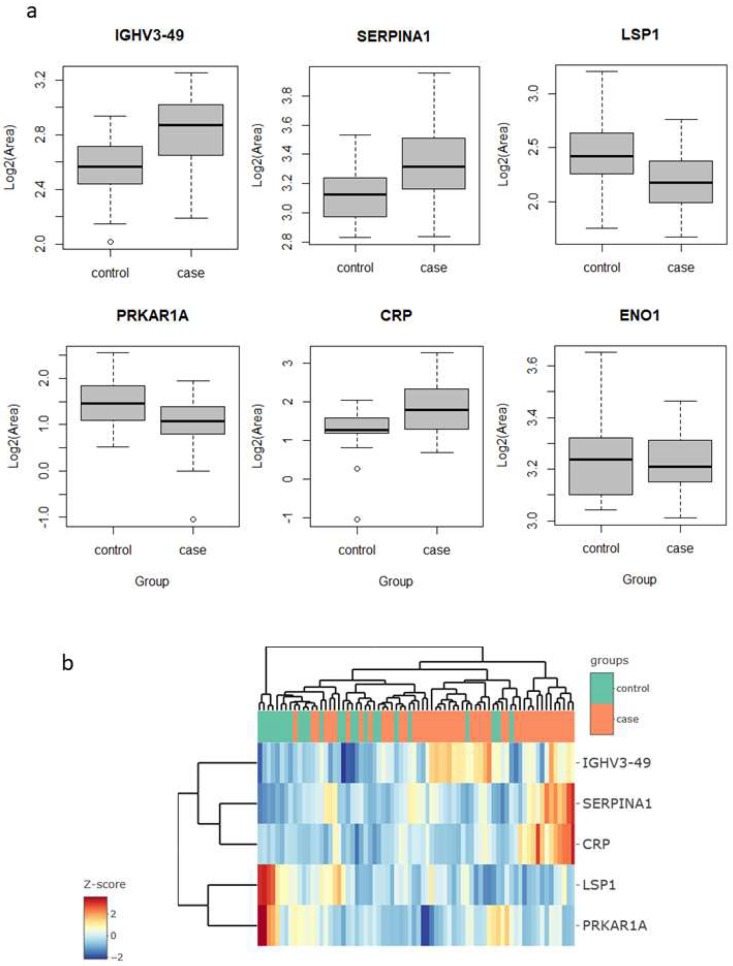
Differential abundance of selected proteins in the sputum proteomes of cases vs. control groups. (**a**) Normalized abundance levels of IGHV3−49, SERPINA1, LSP1, PRKAR1A, CRP, and ENO1. (**b**) Heatmap of the five proteins showing differential abundance in sputum. IGHV3−49, immunoglobulin heavy variable 3−49; SERPINA1, serpin family A member 1; LSP1, lymphocyte specific protein 1; PRKAR1A, protein kinase cAMP-dependent type I regulatory subunit Alpha; CRP, C−reactive protein and protein kinase; ENO1, enolase 1.

The output from Spectronaut was further analyzed in the amica platform. Proteins quantified coming from the contaminant fasta database were excluded from the DE test (10 proteins in total). The output from amica is compiled in Supplementary Table S3. When comparing cases vs. controls using the limma DE test, five proteins showed a significant change in sputum levels, with an adjusted *p*-value below 0.05 and a fold change above 1.5 (in either direction). These proteins were immunoglobulin heavy variable 3–49 (IGHV3-49), C-reactive protein (CRP) and serpin family A member 1 (SERPINA1), which were upregulated in the lung cancer group, and protein kinase cAMP-dependent type I regulatory subunit Alpha (PRKAR1A) and lymphocyte specific protein 1 (LSP1), which were downregulated (Figure 1a).

Interestingly, four of these five proteins are related to inflammatory and immune processes. CRP is engaged in complement activation and amplification. It has defense-related functions based on its ability to recognize pathogens and damaged cells and initiate their elimination by interacting with humoral and cellular effectors in the blood. Consequently, the level of this protein in plasma increases greatly during acute phase response to tissue injury, infection, or other inflammatory stimuli [30]. For instance, the elevated expression of CRP is associated with severe acute respiratory syndrome SARS-CoV-2 pneumonia [31]. Additionally, it has recently shown to be associated with chronic inflammations [32]. In our findings, the increase in their levels (lung cancer to control fold change 2.9) could, therefore, be explained as a consequence of inflammation and/or lung damage caused by lung cancer. SERPINA1 encodes alpha-1-antitrypsin (AAT), which is a serine protease whose targets include elastase, plasmin, thrombin, and plasminogen activator. As CRP, AAT is an acute phase protein. Defects in this protein are associated with chronic obstructive pulmonary disease (COPD) and emphysema, and it has been described as playing an active role in the pathogenesis of cancer (e.g., migration and apoptosis resistance) and the related inflammatory reaction [33]. In this sense, higher serum AAT levels have been associated with worse prognosis in lung cancer [34], and in our study, we found it to be more abundant in sputum in the cases group (fold change 3.0), which reinforces the potential for its use as a marker in lung cancer. However, the mechanisms behind the regulation of AAT expression in lung cancer are still unclear, so further research is needed to determine whether it can be used as a diagnostic marker. IGHV3-49 is a region of the variable domain of Ig heavy chains that participate in antigen recognition. We found it in sputum with levels 2.2-times higher in lung cancer than in the control group. This could be an indication that the immune response, phagocytosis, and the complement classical pathway are being activated [35].

LSP1 is an intracellular F-actin binding protein. This protein is expressed in lymphocytes, neutrophils, macrophages, and endothelium and may regulate neutrophil activation. Although neutrophils have been described as crucial mediators in the development of some tumors, the complete role of neutrophils in cancer biology is still contradictory [36]. On the one hand, its pro-tumorigenic action has been demonstrated by promoting an inflammatory environment that enhances tumor growth. On the other hand, several studies have demonstrated its cytotoxic activity against different types of tumors, even reporting complete tumor regression after neutrophil migration and activation in rats [37]. In contrast, our results, lower LSP1 levels in the lung cancer group (fold change 0.47), could indicate a decrease in neutrophil activation rather than an increase in either pro- or anti-inflammatory activity. PRKAR1A is a regulatory subunit of protein kinase A, which is involved in cAMP signaling in cells by the phosphorylation of different target proteins. It has been described as a tumor-suppressor gene, showing inactivation and decreased expression in thyroid cancer [38] and other endocrine and adrenocortical tumors [39]. For the first time, in this study, it was found to be decreased in the sputum of lung cancer patients (fold change 0.57). Although strongly significant in the limma test, the levels of these proteins alone do not allow a clear separation of the samples of the two groups, cases vs. controls, as seen in the heatmap clustering (Figure 1b).

In previous studies in sputum, enolase 1 (ENO1) and matrix metalloproteinase 2 (MMP2) proteins have been reported as possible lung cancer markers. Yu et al. [21] found higher levels of ENO1 in the sputum of patients compared to cancer-free individuals, as measured using Western blotting. They evaluated diagnostic performance with ELISA in a set of 35 cases and 36 controls, reporting a sensitivity of 58% for a specificity of 80%, with an AUC value to separate the two groups of 0.71. In our study, ENO1 was one of the proteins quantified, but did not result in a significant change in abundance (fold change 0.88, *p*-value 0.42) (Figure 1a). Ali-Labib et al. [23], using a commercial ELISA kit, described an increase in serum and sputum levels of MMP2 in lung cancer (*n* = 32) in comparison with the benign pulmonary diseases group (*n* = 20) and a healthy group (*n* = 38). They reported high sensitivity and specificity values. In our study, we were unable to quantify or detect MMP2 in our sputum samples. We were able to quantify other matrix metalloproteinases, which resulted in no statistically significant change in abundance: MMP8 (fold change 0.67, *p*-value 0.12), MMP9 (fold change 0.64, *p*-value 0.11), and MMP10 (fold change 0.91, *p*-value 0.70).

### 2.2. Functional Analysis

As mentioned above, four of these five proteins are related to inflammatory and immune processes. However, to explore the changes at the biological level overall, we performed functional analysis of the quantitative results with STRING and iPathwayGuide platforms, including pathway analysis, gene-ontology analysis and network analysis. We used as input all the protein groups quantified in our analysis, selecting a less stringent threshold, fold change above 1.5 (in either direction) and *p*-value < 0.01. This resulted in 33 DE genes, 6% (thus between the 5% and 10% recommended by iPathwayGuide) of the number of genes in the reference set (considering all the proteins measured as background). In our dataset, this corresponded to an FDR of 0.15, i.e., 15% of genes resulting in DE simply by chance, although we are more confident that we did not exclude proteins presenting real sputum abundance changes. When filtering with these thresholds, 16 proteins were more abundant in lung cancer patients, and 17 proteins in non-lung cancer controls (the five proteins listed above in 2.1 showed the most significant/extreme changes) (Supplementary Table S3).

**Figure 2:**
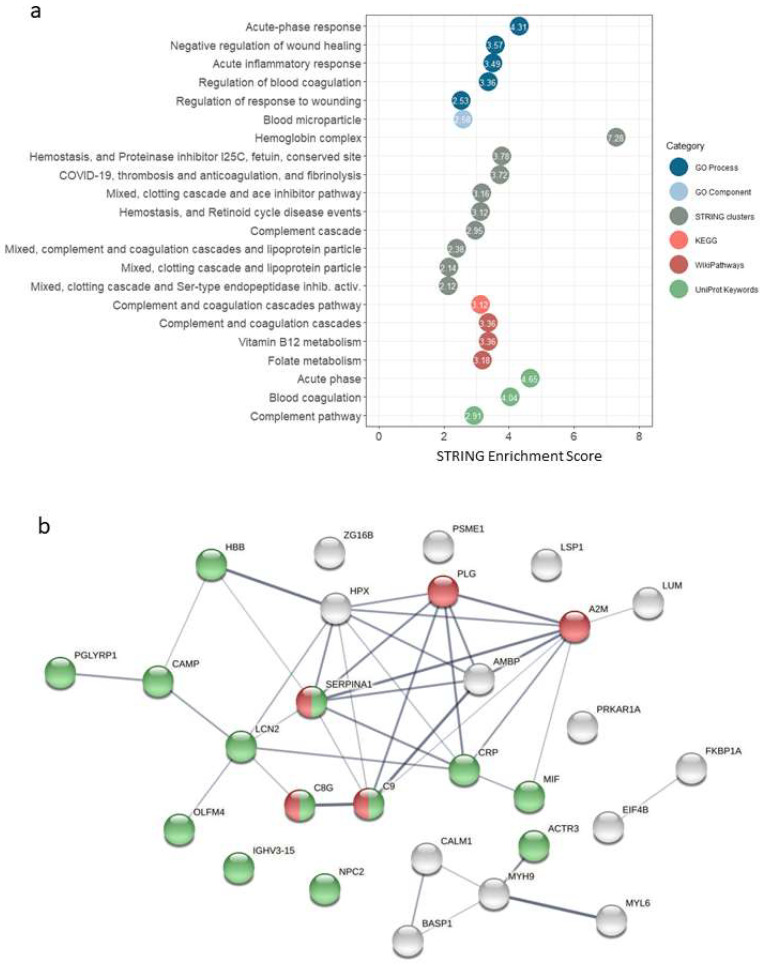
Functional analysis of the quantitative results. (**a**) Functional analysis in STRING, showing enriched terms (FDR < 0.01) for different categories (GO Process, Go Component, STRING clusters, KEGG, WikiPathways and UniProt Keywords). (**b**) STRING interaction network for the mapped differentially abundance proteins. Nodes represent proteins, edges represent protein–protein interactions (line thickness indicates the strength of data support for each interaction). Highlighted nodes: complement and coagulation cascades (KEGG pathway hsa04610) in red; immune response (GO Biological Process GO:0006955) in green.

The results of the functional analysis in STRING, for different categories (GO Process, Go Component, STRING Clusters, KEGG, WikiPathways and UniProtKeywords), are shown in Figure 2a and Supplementary Table S4. We found enrichment of terms (FDR stringency high, 0.01) related to acute-phase response, inflammatory response, complement and coagulation cascades. Figure 2b shows the interaction network for the selected proteins (fold change > 1.5 and *p*-value < 0.01). The over-representation of proteins related to immune response processes could be observed, as well as five proteins of the complement and coagulation cascades pathway occupying a central position in terms of evidence of interactions.

**Figure 3:**
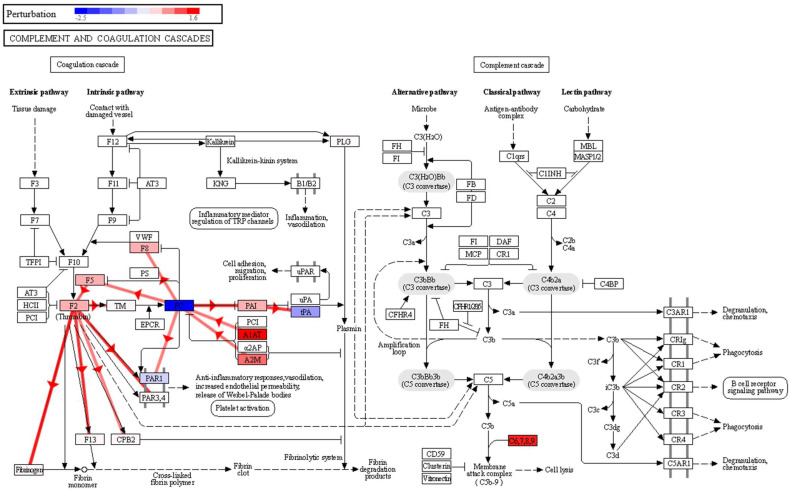
Effect of lung cancer alteration on the sputum proteome, on the complement and coagulation cascades pathway (KEGG:05171), highlighting protein perturbation according to our quantification results and showing coherent cascades.

iPathwayGuide implements a different approach, based on systems biology, to identify significantly impacted pathways. In this case, in addition to the number of DE genes (i.e., overrepresentation analysis), it takes into account other key features, such as the magnitude of the change in the level of each protein and the topological information (position, direction, role and relationships of each gene/protein in a pathway) [40]. With this tool, three pathways were observed to be significantly affected at the pathway level (Supplementary Table S5): complement and coagulation cascades (KEGG: 04610, *p*-value 0.006), coronavirus disease—COVID-19 (KEGG: 05171, *p*-value 0.017), and vascular smooth muscle contraction (KEGG: 04270, *p*-value 0.020). The complement system is a proteolytic cascade in blood plasma and a mediator of innate immunity through the recruitment of inflammatory and immunocompetent cells. The complement upregulation observed in the sputum samples of the lung cancer patient group may confirm a potential link with inflammatory processes and also in cell lysis through the membrane attack complex (Figure 3). This complex is formed by the proteins C5, C6, C7, C8A-B-G and C9, and in our data, in addition to significantly overexpressed C8G and C9, we found C5, C6, C7 and C8B with fold changes showing overexpression in the lung cancer group, although with non-significant *p*-values. The observed effect on the complement and coagulation cascades is in agreement with what we found in our previous study in BALF [12], where we also found a significant impact on this pathway, with seven proteins affected, namely, FG, A2M, PLG, HF1, C5, CQ and C4BP, in higher abundance in BALF in the group of lung cancer patients. In this study, we also found that A2M and PLG had a higher abundance in lung cancer, but this time in sputum. Therefore, the impairment of this pathway could be used as an indicator of lung cancer in both BALF and sputum, which warrants a more detailed and targeted study.

In the coronavirus disease–COVID-19 pathway (*p*-value 0.017), two proteins are overexpressed, C8G and C9. Although this was found to be significant in our analysis, the impact is actually on the complement cascade area of this pathway (Supplementary Figure S1), again evidencing complement activation and inflammation in the group of lung cancer patients. Indeed, the diagram of this pathway shows a possible perturbation of macrophage activation and cytokine release (starting with IL-6), and cell damage through the activation of the membrane attack complex, which again leads to the enhancement of the inflammatory response. In the same vein, IL6 activation would be an upstream regulator of three proteins found to be overexpressed in our data and related to complement cascade and inflammation (A2M, SERPINA1 and CRP) (Supplementary Figure S2). IL6 is produced at sites of acute and chronic inflammation, where it is secreted into the serum and induces a transcriptional inflammatory response, although we have no IL6 data in the sputum dataset to confirm that this mechanism occurs in lung cancer patients.

Regarding the vascular smooth muscle contraction pathway, we found a decrease in lung cancer patients of calmodulin (calmodulin 1, CALM1) and myosin (myosin light chain 6, MYL6, and myosin heavy chain 9, MYH9) subunits (Supplementary Figure S3). Calmodulin is involved in phosphorylation-based signaling pathways, and has been described as playing a role in tumor cell migration, invasiveness and metastasis [41]. Moreover, in the complement and coagulation cascades pathway (Figure 3), a negative perturbation of Proteinase-activated receptor 1 (PAR1) was observed, as a result of the inhibition of vitamin K-dependent protein C (PC). This could be a possible link between the complement and coagulation cascades and the impairment observed in the vascular smooth muscle contraction pathway. Thus, a strong increase in A1AT and A2M expression, observed in our data, would inhibit PC, which exerts a protective effect on the endothelial cell barrier function [42]. Lowering PC would decrease PAR1 activation, which is key in platelet activation [43]. By negatively regulating platelet activation, it would affect the anti-inflammatory response, vasodilation, and endothelial permeability.

### 2.3. Feature Selection

When selecting informative variables (e.g., feature selection), i.e., selecting a panel of proteins from the dataset that allows us to discriminate/classify between different groups, and/or predict the outcome status of a patient, it is important to detect correlated variables, in order to reduce the high dimensionality inherent to high-throughput biological data. Although statistical tests (e.g., *t*-test and limma) are commonly used to identify differentially expressed genes or proteins, they are often sensitive to highly correlated variables, which might be neglected in the variable selection process. Additionally, machine learning algorithms (e.g., support vector machines and random forest) are also frequently applied for predictive purposes. A third option, especially useful in the case of multiple highly correlated variables, is to use multivariate exploratory approaches, such as partial least squares regression (PLS), linear discriminant analysis (LDA), or the more recent sSPLS-DA. PLS-DA is a linear, multivariate model which seeks components that best separate the sample groups, while sSPLS-DA performs variable selection and classification in a single step. It has been shown to work well for informative variable selection, classification and prediction in a multi-class classification scheme [44]. Here, we use sPLS-DA in the R package mixOmics, specifically designed for the analysis of large biological datasets.

**Figure 4:**
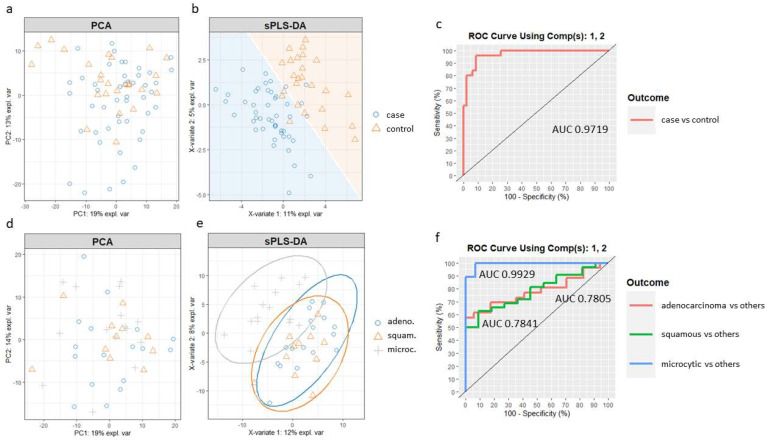
Feature selection with sPLS−DA in mixOmics for (**a**–**c**) the lung cancer vs. control groups comparison and (**d**–**f**) adenocarcinoma, squamous and microcytic groups using one-vs-all comparisons. (**a**,**d**) Preliminary analysis with PCA; (**b**,**e**) sPLS−DA sample plot with (**b**) sample prediction area or (**e**) confidence ellipse plots; (**c**,**f**) ROC curves and AUC.

First, we created a model for the lung cancer case vs. control comparison. The R script used is available in the Supplementary Materials (Supplementary Script S1). Principal component analysis (PCA) was first applied to assess the potential improvement that sPLS-DA could enable. PCA showed no separation between the case and control samples (Figure 4a). Then, we built the sPLS-DA model. The number of components and features per component to use in the sPLS-DA model was tuned by mixOmics using a ten-fold, cross-validation procedure repeated 50 times, following the mixOmics guidelines. Performance was measured via the Balanced Error Rate (BER). The BER is appropriate in the case of an unbalanced number of samples per class as it calculates the average proportion of incorrectly classified samples in each class, weighted by the number of samples in each class. Therefore, the BER is less biased towards majority classes during the performance assessment [45]. The centroids distance metric was used, since it provided the best classification accuracy. The tuning process resulted in a model with two components and a molecular profile comprising 30 and 20 features selected for the first two components (Supplementary Table S6). Figure 4b shows the sample plot for those first two components, depicting the prediction background generated by the samples. Although there is some overlap, it can be seen that the model is able to separate the two groups of individuals with good accuracy, outperforming PCA. Furthermore, ROC analysis (Figure 4c) suggested that the optimized sPLS-DA model can discriminate lung cancer patients from controls with a high rate of true positives and a low rate of false positives (AUC of 0.97).

A new sPLS-DA model was created to differentiate patients from the three main cancer groups, adenocarcinoma, squamous and microcytic, using a subset of the respective samples. In this case, when compared to the PCA (Figure 4d), an sPLS-DA model including the first two components improved the separation of the microcytic cases, although squamous and adenocarcinoma samples still overlapped (Figure 4e). The ROC curves and AUC of the final sPLS-DA model were also calculated using one-vs-all comparisons (Figure 4f). The model including the first two components led to a remarkable classification accuracy for the microcytic cancer patients (AUC of 0.99), while the model was less well suited to distinguish subjects in the epidermoid and adenocarcinoma groups (AUC of 0.78 for both).

### 2.4. Targeted Analysis of SERPINA1

Using Skyline software, we developed a targeted assay for six peptides from the protein SERPINA1, which we had previously found to be upregulated in the sputum of the cancer group and which showed the higher fold change of the significant reported proteins (lung cancer to control fold change 3.0). We used also Skyline to load and process the targeted runs from 24 samples, and to obtain the quantitative info for the transitions monitored (the six top transitions per targeted peptide). The final list with the peptides, precursors and transitions used for protein quantification is shown in Supplementary Table S7. Chromatograms were manually curated for all precursors in every sample. Retention times and relative intensities of the transitions within a precursor were verified for each precursor. Variability for retention times was low (ranging from 0.62 to 2.14% coefficient of variation) and the transition relative intensities were homogenous, showing the reproducibility of the assay. This exploratory data analysis in Skyline is shown in Supplementary Figure S4.

The adjusted (Benjamini–Hochberg) *p*-values for the group comparison (cancer vs. controls) verified that the protein and all the six peptides were significantly more abundant in the cancer than in the control group. Supplementary Table S8 shows the fold changes and adjusted *p*-values as reported by Skyline. The targeted analysis for SERPINA1 showed a fold change of 4.53 (lung cancer to control), and an adjusted *p*-value of 0.0017. This fold change is even higher than that previously reported in the discovery analysis (fold change of 3.0).

## 3. Material and Methods

**Figure 5:**
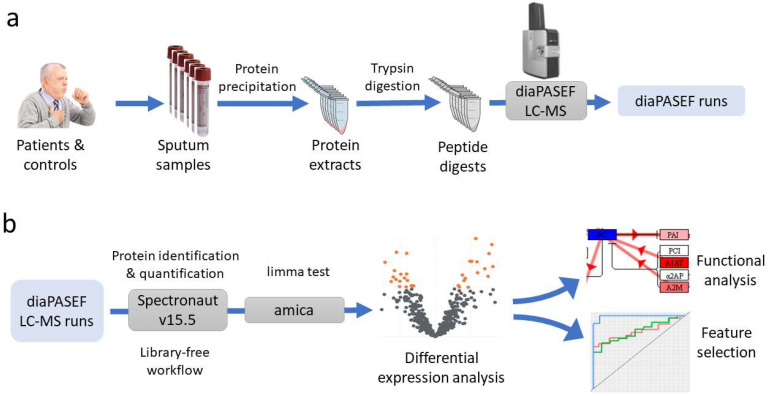
Proteomics workflow. (**a**) Sample preparation and LC-MS acquisition using diaPASEF, and (**b**) data analysis.

The proteomics workflow followed is summarized in Figure 5 and detailed in the following sections.

### 3.1. Patients and Sputum Sample Collection

The individuals included in the study were recruited from the patients of the Pneumology Department of the Reina Sofía University Hospital (Córdoba, Spain). For the group of patients with lung cancer, patients diagnosed by PET or PET-CT and who were less than 75 years old were included. The lung cancer diagnosis included clinical tests based on fine needle biopsy, bronchoscopy, video-assisted thoracoscopy and subsequent cytohistology confirmation. The anatomical pathology service performed the cytohistological tests to determine the histological type. The present accepted guidelines for pathological and staging diagnosis of lung cancer were used [46]. For the control group of non-lung cancer patients, we included individuals aged 55–75 years; smokers or ex-smokers within the last 15 years with a cumulative consumption of >30 packs/year; and the absence of symptoms suggestive of malignancy, including hemoptotic expectoration, change in cough characteristics or constitutional syndrome, as well as the absence of findings suggestive of malignancy upon chest CT. Subjects who were older than 75 years and those in whom it was not possible to establish the diagnosis of malignancy or coexistence of extrapulmonary neoplasia in the last 5 years were excluded. Patients with significant comorbidity, such as severe organ disease with a negative impact on prognosis or preventing the application of the study protocol, were also excluded. All the patients provided informed consent. The study was performed according to the principles of the Declaration of Helsinki, aligning with the European Union regulation 2016/679, and was approved by the Research Ethics Committee of Cadiz.

After washing with physiological saline aerosol and gargling with water, coughing was induced in each patient to produce sputum, which was collected in a container and chilled. To 2 mL of sputum, 8 mL of phosphate-buffered saline, 200 μL of protease inhibitor cocktail, and 200 μL of 100 mM DTT were added. After incubation at 37 °C for 10 min, samples were shaken for 10 min, and centrifuged for 10 min to separate the cell debris. The supernatant was collected and filtered over 1.5 mL tubes, centrifuged for 10 min, and the supernatant was aliquoted and stored at −80 °C until processing and analysis.

### 3.2. Sample Preparation

One aliquot of each sample was subjected to protein precipitation with cold acetone. Protein pellets were resuspended in 50 μL of 0.2% RapiGest (Waters, Milford, MA, USA) in 50 mM ammonium bicarbonate. The protein content was measured in a Qubit fluorimeter (Thermo Fisher Scientific, Waltham, MA, USA) using the Qubit Protein Assay kit (Thermo Fisher Scientific), and 40 μg of each sample was digested with trypsin as in Ortea et al., 2018 [47]. In brief, after incubation with 5 mM DTT (30 min, 60 °C) and iodoacetamide (30 min, room temperature), protein samples were digested in two steps (1:40 trypsin-to-protein ratio, 2 h plus 15 h incubation at 37 °C). RapiGest was precipitated by incubation with 0.5% TFA (1 h, 37 °C) and centrifugation. Peptide digests were then diluted with 0.2% TFA to 100 ng/μL of equivalent protein content.

### 3.3. Nano-Liquid Chromatography—Mass Spectrometry (nLC-MS) Acquisition

Samples (2 μL, 200 ng protein digest on column) were analyzed on a timsTOF Pro (Bruker, Billerica, MA, USA) Q-TOF mass spectrometer coupled to a nanoElute (Bruker) liquid chromatography (LC) system. A C18 Aurora Series UHPLC emitter column (250 mm × 75 μm id, 1.6 μm, 120 Å pore size) (IonOpticks, Fitzroy, Australia) was used for all the analyses, using a trap-elute configuration with an Acclaim PepMap C18 (5 mm, 300 μm id, 5 μm particle diameter, 100 Å pore size) trap cartridge (Thermo Fisher Scientific). The gradient and LC parameters were the same for all the analyses: peptides were eluted at a 45 min gradient from 5 to 30% B (from 5 to 25% B in 40 min; from 25 to 30% B in 5 min), plus 5 min to increase B from 30% to 80% and 7 min of column cleaning (80% B), with A denoting water and B denoting ACN, both with 0.1% FA. The chromatography flow rate was 300 nL/min, and the column oven was set to 50 °C. As the peptides eluted from the chromatography to the mass spectrometer, they were ionized in a Captive nano-electrospray source (Bruker) at 1500 V.

Samples were run using a diaPASEF acquisition method consisting of 12 cycles including a total of 32 mass width windows (27.2 Da width, from 380 to 1250 Da) with 2 mobility windows each, making a total of 64 windows covering the ion mobility range (1/K_0_) from 0.61 to 1.50 V s/cm^2^. These windows were optimized by applying the Window Editor utility from the instrument control software (timsControl, Bruker) using a DDA-PASEF run previously acquired from a pool of the analyzed samples. In brief, this utility loaded the run and represented its ion density in the *m/z* and ion mobility ranges (i.e., the mobility heatmap), so the diaPASEF window coverage could be adjusted to ensure optimum coverage, and the window settings were calculated. The collision energy was programmed as a function of ion mobility, following a straight line from 20 eV for 1/K_0_ of 0.6 V s/cm^2^ to 59 eV for 1/K_0_ of 1.6 V s/cm^2^. The TIMS elution voltage was linearly calibrated to obtain 1/K_0_ ratios using three ions from the ESI-L Tuning Mix (Agilent, Santa Clara, CA, USA) (*m/z* 622, 922, 1222) before each run, by applying the ‘Automatic calibration’ utility in the control software (timsControl, Bruker).

### 3.4. Data Analysis

The directDIA workflow in Spectronaut version 15.5 (Biognosys, Schlieren, Switzerland) was used to process the diaPASEF LC–MS runs with no need to build a previous library from DDA runs. In brief, this processing consisted of two sequential steps, a database search using Pulsar Spectronaut’s search engine, and DIA analysis. A SwissProt human protein reference database (UP000005640 isoform fasta database, downloaded on 8 July 2021, containing 42,351 sequences) was used for the search in Pulsar, together with a fasta file containing 112 common contaminant sequences. The default factory settings were used for the Pulsar search and library generation, including Trypsin/P as the enzyme; specific digest type; a 7–52 peptide length range; up to two missed cleavages allowed; the oxidation of Met and acetylation of Protein N-t as variable modifications; carbamidomethyl of Cys as fixed modification, and 1% FDR for PSM, peptide and protein group identification. The generated spectral library was then used by Spectronaut for DIA analysis, that is, extracting the quantitative information from the diaPASEF runs. The default factory settings were used, except for the calibration MS1 and MS2 mass tolerances, which were set to 20 ppm; proteotypicity filter was set to ‘only protein group specific’. An automatic cross-run normalization strategy (e.g., local normalization) was followed, and the MaxLFQ method was used for protein quantification. The quantity was determined at the MS2 level using the area of extracted chromatogram traces.

For differential expression (DE) analysis, the output from Spectronaut was further analyzed in the amica platform [48]. LFQ intensities of quantified proteins were log2-transformed and quantile normalized, and missing values were imputed from a normal distribution downshifted 1.8 standard deviations from the mean with a width of 0.3 standard deviations (default parameters). Differential expression analysis was performed using limma [49]. For functional analysis, the result from differential expression analysis was further analyzed in STRING version 11.5 (https://string-db.org/ (accessed on 15 April 2022)) [50] and iPathwayGuide version 2201 (Advaita Corporation, Plymouth, MI, USA). STRING was used for interaction network analysis and for analyzing the functional enrichments in the network. iPathwayGuide was used for analyzing the significantly impacted pathways, in the context of pathways obtained from the Kyoto Encyclopedia of Genes and Genomes (KEGG) database (Release 100.0+/11-12, 21 November). Feature selection was performed by sparse partial least squares discriminant analysis (sPLS-DA) using mixOmics R package version 6.1.1 [45].

### 3.5. Targeted Analysis of SERPINA1

For verification of the followed discovery approach and results, Skyline (version 21.2.0.568) [51] was used to build up a targeted method for monitoring one of the proteins previously found as changing in abundance (protein SERPINA1). Only precursor *m/z*’s from tryptic theoretical peptides were included in the assay. Peptide settings excluded peptides with missed cleavages, peptides below 7 or above 26 amino acid length, and peptides containing methionine or cysteine. Initial set of transitions were filtered from ion 3 to last ion, *y* and *b* ion types, and precursor charge 2+. A total of 24 samples were run for performing these targeted analyses, using the same high-resolution Q-TOF and LC gradient (45 min) as specified above. MS1 filtering was set to 3 centroided TOF MS peaks, with mass accuracy of 20 ppm, and instrument minimum and maximum *m/z* was set to 50 and 1800, respectively.

Subsequently, the LC-MS runs for the 24 samples for targeting protein SERPINA1, monitoring six peptides, were loaded into Skyline, and the area under the curve for the selected transitions from each peptide was calculated. For those precursors with more than six transitions, only the six highest intensity transitions were selected for quantification. Measures were normalized to the total ion current, and the sum of transition areas was selected as the summary method. Skyline was also used for performing the statistical group comparison, calculating fold changes and performing a *t*-test at both protein and peptide levels, and adjusting *p*-values for multiple hypothesis testing with the Benjamini–Hochberg correction.

## 4. Conclusions

In the present study, we showed how LC–MS working in the recently developed diaPASEF mode can be used to detect protein changes that may represent potential biomarkers in sputum samples and, ultimately, discriminate patients from controls and even help to differentiate between different cancer subtypes. We detected in the sputum proteome of the lung cancer group an activation of inflammation, observed from the alteration of pathways and processes related to acute-phase, complement cascade, and immune response. Furthermore, by applying feature selection, we demonstrated how a correct selection of components and features in an sPLS-DA model allows us to separate the samples studied according to the group of origin with high levels of sensitivity and specificity.

A number of potential markers with the ability to differentiate between lung cancer patients and healthy controls are, therefore, proposed, which, after validation in further studies, could be incorporated into the diagnostic algorithm in the at-risk population. Although the panel of potential biomarkers presented needs further validation, the prioritization that the feature selection process has provided could help speed up the biomarker development process by focusing on which proteins to target in a larger number of individuals. In addition, obtaining digital proteome maps of sputum samples, obtained through MS with diaPASEF acquisition, and comparing them with chemometric tools, such as sPLS-DA, constitutes a useful approach in the classification of individuals. Implementing a tool of low invasiveness that informs us about which patients have a higher probability of developing lung cancer, and can, therefore, be incorporated into screening and/or diagnostic programs, represents an advance applicable to the healthcare system and with obvious repercussions for current clinical practice guidelines.

## Data Availability

The mass spectrometry proteomics data have been deposited to the ProteomeXchange Consortium via the PRIDE partner repository with the dataset identifier PXD032269.

## Supplementary Materials

The following supporting information can be downloaded at: https://www.mdpi.com/article/10.3390/ijms23158737/s1.
