# Supplementary material for: diaPASEF Proteomics and Feature Selection for the Description of Sputum Proteome Profiles in a Cohort of Different Subtypes of Lung Cancer Patients and Controls
Source: Int J Mol Sci. 2022 Aug 5;23(15):8737. doi: 10.3390/ijms23158737 (PMC9369298; doi:10.3390/ijms23158737)

SUPPLEMENTARY MATERIAL

Supplementary Table S1. Windows scheme used for the diaPASEF acquisition. IM, ion mobility.

MS Level	TIMS Scan	Start Mass [m/z]	End Mass [m/z]	Start IM [1/K0]	End IM [1/K0]
MS1	0	na	na	na	na
diaPASEF	1	1223	1250	1.38	1.50
diaPASEF	1	1060	1087	1.25	1.37
diaPASEF	1	897	924	1.13	1.25
diaPASEF	1	733	761	1.00	1.12
diaPASEF	1	570	597	0.88	1.00
diaPASEF	1	407	434	0.75	0.87
diaPASEF	1	380	407	0.61	0.73
diaPASEF	2	1196	1223	1.36	1.48
diaPASEF	2	1033	1060	1.23	1.35
diaPASEF	2	869	897	1.11	1.23
diaPASEF	2	706	733	0.98	1.10
diaPASEF	2	543	570	0.86	0.98
diaPASEF	2	380	407	0.73	0.85
diaPASEF	3	1087	1114	1.27	1.40
diaPASEF	3	924	951	1.15	1.27
diaPASEF	3	761	788	1.02	1.15
diaPASEF	3	597	625	0.90	1.02
diaPASEF	3	434	462	0.77	0.90
diaPASEF	3	407	434	0.63	0.75
diaPASEF	4	1114	1141	1.29	1.42
diaPASEF	4	951	978	1.17	1.29
diaPASEF	4	788	815	1.04	1.17
diaPASEF	4	625	652	0.92	1.04
diaPASEF	4	462	489	0.79	0.92
diaPASEF	4	434	462	0.65	0.77
diaPASEF	5	1141	1168	1.32	1.44
diaPASEF	5	978	1005	1.19	1.31
diaPASEF	5	815	842	1.07	1.19
diaPASEF	5	652	679	0.94	1.06
diaPASEF	5	489	516	0.82	0.94
diaPASEF	5	462	489	0.67	0.79
diaPASEF	6	1168	1196	1.34	1.46
diaPASEF	6	1005	1033	1.21	1.33
diaPASEF	6	842	869	1.09	1.21
diaPASEF	6	679	706	0.96	1.08
diaPASEF	6	516	543	0.84	0.96
diaPASEF	6	489	516	0.69	0.82
diaPASEF	7	1168	1196	1.21	1.34
diaPASEF	7	1005	1033	1.09	1.21
diaPASEF	7	842	869	0.96	1.09
diaPASEF	7	679	706	0.84	0.96
diaPASEF	7	516	543	0.71	0.84
diaPASEF	8	1196	1223	1.24	1.36
diaPASEF	8	1033	1060	1.11	1.23
diaPASEF	8	869	897	0.99	1.11
diaPASEF	8	706	733	0.86	0.98
diaPASEF	8	543	570	0.74	0.86
diaPASEF	9	1223	1250	1.26	1.38
diaPASEF	9	1060	1087	1.13	1.25
diaPASEF	9	897	924	1.01	1.13
diaPASEF	9	733	761	0.88	1.00
diaPASEF	9	570	597	0.76	0.88
diaPASEF	10	1087	1114	1.15	1.27
diaPASEF	10	924	951	1.03	1.15
diaPASEF	10	761	788	0.90	1.02
diaPASEF	10	597	625	0.78	0.90
diaPASEF	11	1114	1141	1.17	1.29
diaPASEF	11	951	978	1.05	1.17
diaPASEF	11	788	815	0.92	1.04
diaPASEF	11	625	652	0.80	0.92
diaPASEF	12	1141	1168	1.19	1.32

diaPASEF	12	978	1005	1.07	1.19
diaPASEF	12	815	842	0.94	1.07
diaPASEF	12	652	679	0.82	0.94

Supplementary Table S2. Quantitation table. A total of 552 protein groups were identified, corresponding to 914 proteins, and 527 protein groups quantified, after processing the diaPASEF runs with the directDIA workflow in Spectronaut v15.5 software.

Supplementary Table S3. Result for the differential expression analysis using limma test in the amica platform.

Supplementary Table S4. Result for the functional enrichment analysis in STRING v11.5 for the categories GO Process, Go Component, STRING Clusters, KEGG, WikiPathways and UniProtKeywords. Only statistically significant terms for each category are shown.

Supplementary Table S5. Result for the pathways impact analysis in iPathwayGuide v22011 software.

Supplementary Table S6. Features selected for the two components included in the optimized sPLS-DA model for the cases (lung cancer patients) vs. controls comparison.

**Component 1**
IGHV3-49; SERPINA1; PRKAR1A; LSP1; CRP; C9; PLG; HPX; FKBP1A; IGLV2-18; C8G; CAMP; ACTR3; ZG16B; LUM; NPC2; MYL6; IGHV3-15; HBB; MIF; PGLYRP1; IGHV3-23; PSME1; LCN2; CALM1; IGHV1-24; IGHV3-7; OLFM4; MYH9; AMBP
**Component 2**
PLBD1; EGFL6; SFTPA2; TUBB4B; CFB; SERPINF1; TALDO1; PYGL; HNRNPD; TTR; RNASET2; ARG1; SERPINA6; G6PD; KRT4; MDH1; RARRES1; CTSC; CAT; BPIFB1

Supplementary Table S7. Targeted analysis. List with the peptides, precursors and transitions that were monitored and quantified by the targeted assay for the protein with the accession sp|P01009|A1AT_HUMAN (SERPINA1).

Peptide sequence	Precursor *m/z*	Precursor charge	Product *m/z*	Fragment ion
QINDYVEK	504.7535	2	880.4411	y7
			767.3570	y6
			653.3141	y5
			538.2871	y4
			375.2238	y3
			862.3941	b7
SASLHLPK	426.7505	2	694.4246	y6
			607.3926	y5
			494.3085	y4
			357.2496	y3
			496.2514	b5
			609.3355	b6

LSITGTYDLK	555.8057	997.5200	y9
		910.4880	y8
		797.4040	y7
		696.3563	y6
		639.3348	y5
		415.2551	b4
SVLGQLGITK	508.31097	829.5142	y8
		716.4301	y7
		659.4087	y6
		531.3500	y5
		418.2660	y4
		361.2445	y3
VFSNGADLSGVTEEAPLK	917.4651	1143.6256	y11
		1030.5415	y10
		943.5095	y9
		787.4196	y7
		428.2867	y4
		357.2496	y3
AVLTIDEK	444.7555	817.4666	y7
		718.3981	y6
		605.3141	y5
		504.26640	y4
		391.1823	y3
		385.2445	b4

Supplementary Table S8. Group comparison (cancer vs controls) results for the SERPINA1 targeted analysis. Fold changes and Benjamini-Hochberg adjusted p-values are shown at protein and peptides levels, as calculated by Skyline software.

Protein	Peptide	Fold change (95% confidence Interval)	Adjusted p-val
sp|P01009|A1AT_HUMAN (SERPINA1)	-	4.53 (1.89-10.89)	0.0017
	QINDYVEK	3.6 (1.68-7.67)	0.0042
	SASLHLPK	5.32 (1.97-14.39)	0.0042
	LSITGTYDLK	5.06 (1.73-14.84)	0.0074
	SVLGQLGITK	5.07 (2.07-12.41)	0.0042
	VFSNGADLSGVTEEAPLK	4.37 (1.57-12.14)	0.0081
	AVLTIDEK	3.64 (1.03-12.78)	0.0447

Supplementary Script S1. R script used for feature selection using sPLS-DA in mixOmics.

Supplementary Figure S1. Effect of lung cancer alterations on the sputum proteome, on the Coranavirus disease - COVID-19 pathway (KEGG: 05171), highlighting protein perturbation according to our quantification results and showing coherent cascades (iPathwayGuide result).

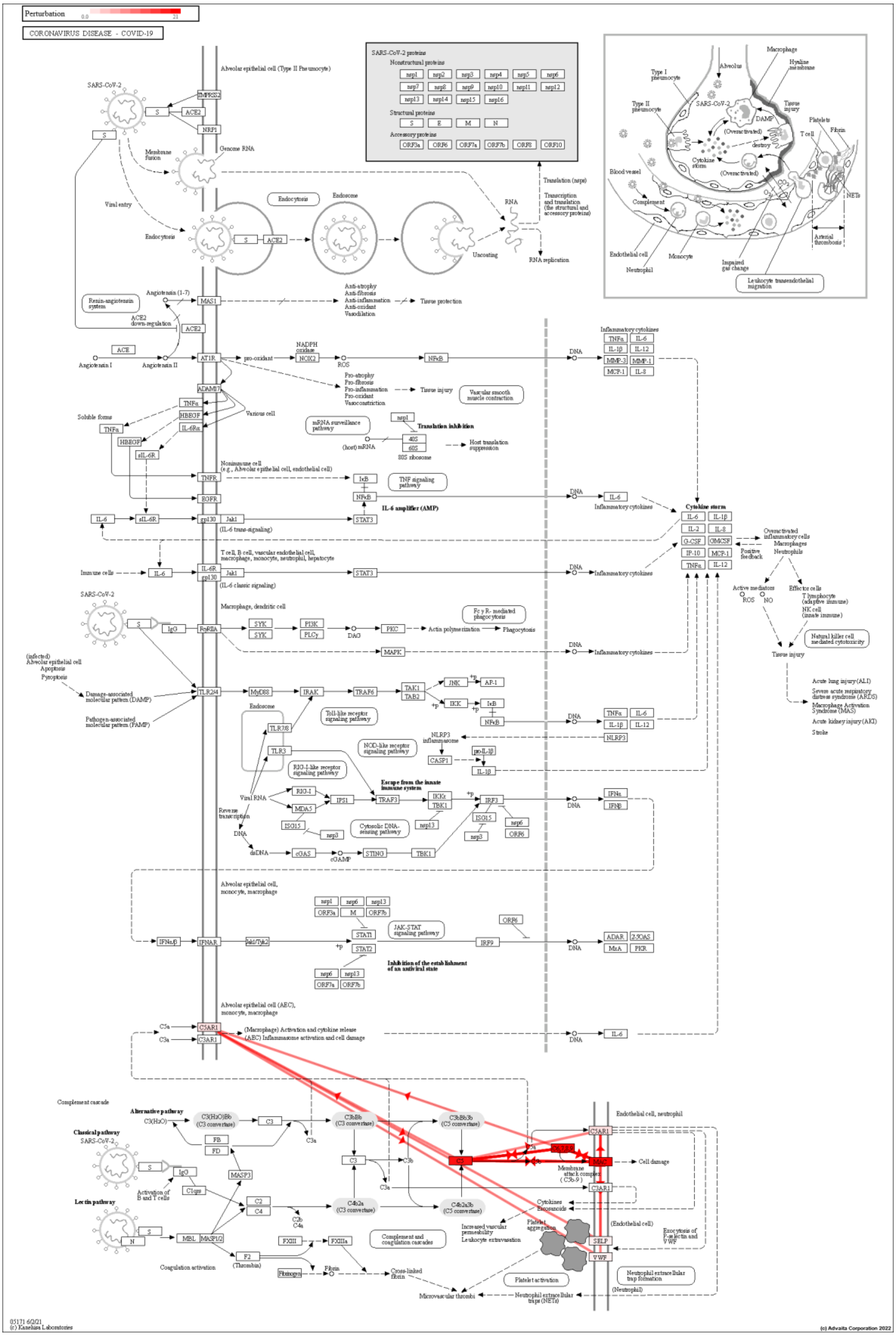

Supplementary Figure S2. iPathwayGuide result from upstream regulators analysis. Interleukin-6 (IL6) is an upstream activator for three proteins (A2M, CRP and SERPINA1) found to be overexpressed in the LC group and related to complement cascade and inflammation.

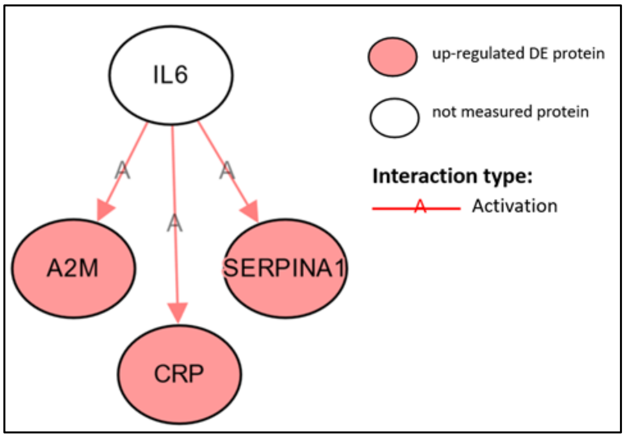

Supplementary Figure S3. Effect of lung cancer alterations on the sputum proteome, on the vascular smooth muscle contraction pathway (KEGG: 04270), highlighting protein perturbation according to our quantification results and showing coherent cascades (iPathwayGuide result).

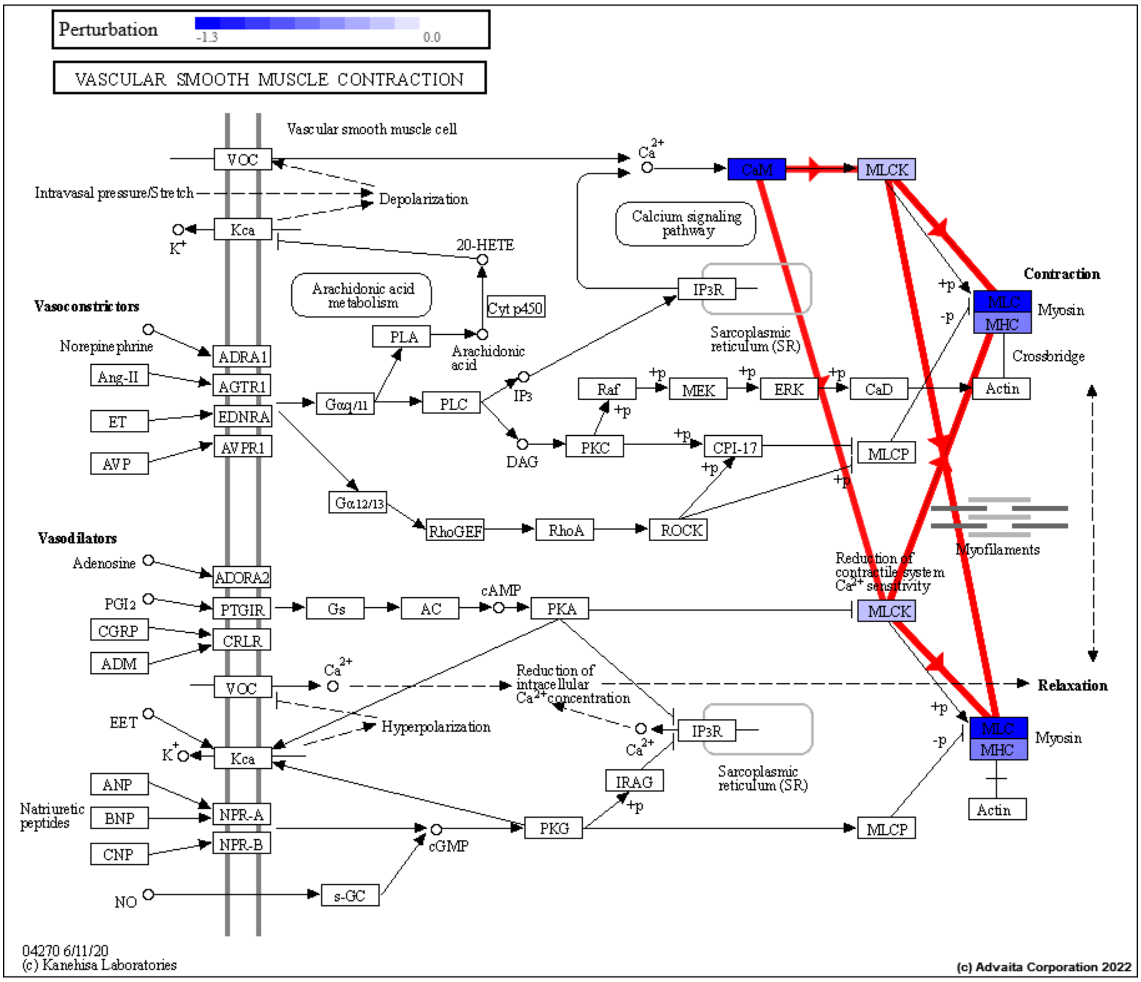

Supplementary Figure S4. Targeted analysis in Skyline. Quality of all targeted peptides and transitions was assessed by (a) visually checking the transition chromatograms, (b) retention times, and (c) transition relative intensities for all the precursors across all runs (as an example, Skyline derived plots are shown for peptide QINDYVEK). (d) The six targeted peptides eluted across the whole chromatogram with low retention time variability. (e) Total ion current normalized peak area for the six targeted peptides grouped by condition. All six peptides are more abundant in the case (cancer) group. (f) and (g) Fold change plots for the individual peptides and the targeted protein, respectively, showing the 95% confidence interval. All of the confidence interval whiskers do not cross the zero line, indicating that at 95% confidence it would be unusual for the observed data to occur by random chance.

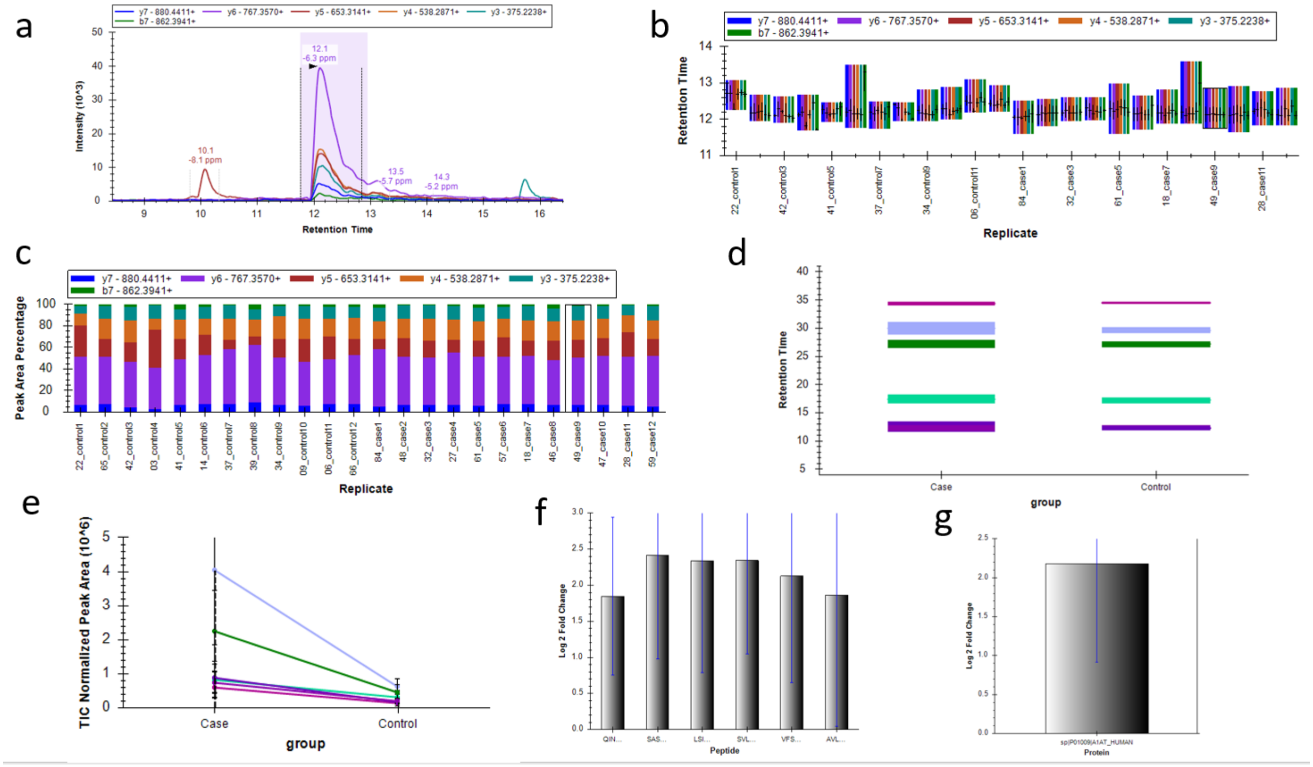

Supplement: Supplementary file 1 [file ijms-23-08737-s001.zip › SUPPLEMENTARY MATERIAL.pdf]
